# Engineering a Human Plasmacytoid Dendritic Cell-Based Vaccine to Prime and Expand Multispecific Viral and Tumor Antigen-Specific T-Cells

**DOI:** 10.3390/vaccines9020141

**Published:** 2021-02-10

**Authors:** Kevin Lenogue, Alexandre Walencik, Karine Laulagnier, Jean-Paul Molens, Houssem Benlalam, Brigitte Dreno, Pierre Coulie, Martin Pule, Laurence Chaperot, Joël Plumas

**Affiliations:** 1 Immunobiology and Immunotherapy of Chronic Diseases, Institute for Advanced Biosciences, INSERM U1209, CNRS UMR 5309, Université Grenoble Alpes, 38700 La Tronche, France; k.lenogue@free.fr (K.L.); alexandre.Walencik@efs.sante.fr (A.W.); jean-paul.molens@efs.sante.fr (J.-P.M.); laurence.chaperot@efs.sante.fr (L.C.); 2 PDC*line Pharma, 38701 Grenoble, France; k.laulagnier@pdc-line-pharma.com; 3 Research and Development Laboratory, Etablissement Français du Sang Auvergne-Rhône-Alpes, 38701 Grenoble, France; 4 HLA Laboratory, Etablissement Français du Sang Centre—Pays de la Loire, 44011 Nantes, France; 5 CRCINA, INSERM, Université d’Angers, Université de Nantes, 44011 Nantes, France; Houssem.Benlalam@univ-nantes.fr; 6 Onco-Dermatology Department, CHU Nantes, CIC 1413, CRCINA, Université de Nantes, 44093 Nantes, France; brigitte.dreno@atlanmed.fr; 7 De Duve Institute, Université Catholique de Louvain, B-1200 Brussels, Belgium; pierre.coulie@uclouvain.be; 8 Cancer Institute, University College London, London WC1E 6BT, UK; martin.pule@ucl.ac.uk

**Keywords:** plasmacytoid dendritic cells, vaccination, immunotherapy, infectious diseases, cancer diseases

## Abstract

Because dendritic cells are crucial to prime and expand antigen-specific CD8^+^ T-cells, several strategies are designed to use them in therapeutic vaccines against infectious diseases or cancer. In this context, off-the-shelf allogeneic dendritic cell-based platforms are more attractive than individualized autologous vaccines tailored to each patient. In the present study, a unique dendritic cell line (PDC*line) platform of plasmacytoid origin, already used to prime and expand antitumor immunity in melanoma patients, was improved thanks to retroviral engineering. We demonstrated that the clinical-grade PDC*line, transduced with genes encoding viral or tumoral whole proteins, efficiently processed and stably presented the transduced antigens in different human leukocyte antigen (HLA) class I contexts. Moreover, the use of polyepitope constructs allowed the presentation of immunogenic peptides and the expansion of specific cytotoxic effectors. We also demonstrated that the addition of the Lysosome-associated membrane protein-1 (LAMP-1) sequence greatly improved the presentation of some peptides. Lastly, thanks to transduction of new HLA molecules, the PDC platform can benefit many patients through the easy addition of matched HLA-I molecules. The demonstration of the effective retroviral transduction of PDC*line cells strengthens and broadens the scope of the PDC*line platform, which can be used in adoptive or active immunotherapy for the treatment of infectious diseases or cancer.

## 1. Introduction

Antigen-specific cytotoxic CD8^+^ T-lymphocytes (CTLs) are potent effectors able to specifically recognize and lyse infected or malignant cells; however, their development through efficient vaccines for the treatment of infectious diseases or cancers remains a challenge. Until now, most of the vaccine strategies aiming to prime or expand these key cellular effectors have failed to bring a significative clinical benefit in humans [1,2,3].

Until now, only one dendritic cell-like cell-based vaccine, Provenge^®^ from Dendreon Pharmaceuticals company, has been approved by the Food and Drug Administration (FDA) for the treatment of advanced prostate cancer patients [4]. Due to their critical role in priming and expansion of CTLs, the use of in vitro differentiated dendritic cells (DCs) and the in vivo targeting of these cells are still active fields of investigation in the development of active immunotherapy strategies, particularly in cancer therapy [5].

In this context, off-the-shelf allogeneic DC-based platforms are more attractive than individualized autologous vaccines tailored to each patient in many aspects (facility, cost, reproducibility of drug product manufacturing, and homogeneity of clinical trials), since the same product is used to treat several patients. There are very few examples of DC platforms. Recently, two reports described the use of allogeneic dendritic cell lines of myeloid origin to induce antigen-specific T-cell responses [6,7]. In one case, DC-like cells were differentiated from the MUTZ3 cell line using a cocktail of several cytokines, chemical agent, and hormone within ten days [6]. In the other case, DC-like cell lines were obtained by cultivating, for three months, the CD3-negative population of activated peripheral blood mononuclear cells (PBMCs) transduced by the tax gene of the Human T cell leukaemia/lymphoma virus type 2 (HTLV2) [7]. These complex manufacturing processes hinder the industrialization of these vaccines, limiting the broadening of their use.

We have developed several human plasmacytoid dendritic cell (PDC) lines from leukemic cells of a patient having a PDC leukaemia for research and development (R&D) applications [8,9] or for clinical applications [10]. One of them, the clinical-grade cell line named ‘PDC*line’ is grown in cell suspension in a synthetic medium without the need for any feeder cell, growth or maturation factor. PDC*line cells are potent professional antigen-presenting cells, able to prime and expand antigen-specific T-cells in both viral and tumour models in vitro and in vivo in an HLA-A*02:01-matched situation [11,12,13,14]. We also demonstrated that PDC*line cells were more potent than moDC to activate and expand potent antitumor T-cells [13]. With this PDC*line, we have developed a new cell-based vaccine approach named GeniusVac/PDC*vac that was first used to boost antitumor immunity in HLA-A*02:01 melanoma patients with a cell therapy product named GeniusVac-Mel4 [10]. The cell product was composed of the irradiated PDC*line cells loaded with four tumour HLA-A*02:01-restricted peptides derived from antigens expressed in melanoma. This first-in-human trial demonstrated that the peptide-loaded PDC*line product was safe and not rejected. Importantly, we demonstrated that this PDC-based product was biologically active and, thus, able to induce the priming and expansion of circulating antitumor CD8^+^ T-cells in patients [10].

The in vitro demonstration of the potency of the PDC*line as antigen-presenting cells, followed by the demonstration of its biological efficiency in vivo, render the strategy very attractive for further clinical developments. Interestingly, both active and adoptive immunotherapy of infectious and cancer diseases could be considered. We wondered whether the PDC*line cells could be optimized by favouring internal processing of peptides by transduction with genes encoding for antigens, and by making it suitable for non-HLA-A*02:01 patients by transduction with genes encoding for new HLA class I molecules.

Several technologies have been developed to engineer human cells for cell therapy [15]. Among them, the use of viral vectors is interesting because genes encoding for antigens are integrated in the host DNA, and proteins are produced continuously. Moreover, such a viral transduction process has been clinically validated to engineer T-cells with chimeric antigen receptor genes in the context of adoptive therapy for lymphoma [16] or glioblastoma [17].

The aim of the study was to demonstrate that PDC*line cells could be used as a wide vaccine platform for the treatment of infectious or tumour diseases thanks to the transduction of genes of interest by viral vectors.

## 2. Materials and Methods

### 2.1. Cells and Peptides

Peripheral blood mononuclear cells (PBMCs) were obtained from healthy donors at the French Blood Bank after giving informed consent (cell collection DC-2008-787 authorized by the French ministry of Health). PBMCs were purified from blood by Ficoll–Hypaque density gradient centrifugation protocol (Eurobio, Les Ulis, France). All PBMCs expressed HLA-A*02:01 except PBMC#2. PBMC#2 and PBMC#3 expressed the HLA-B*07:02 molecule. The human HLA-A*02:01 plasmacytoid dendritic cell line (PDC*line) was generated from a patient having a PDC leukaemia [10]. Leukemic cells were engrafted in an immunodeficient mouse, and the resulting tumour was dilacerated, cultured, and maintained in X-vivo 15 medium (Lonza, Basel, Switzerland) to generate a cell bank. The HLA class I typing of PDC*line cells is the following: A*02:01, B*07:02, B*44:02, C*05:01, and C*07:02. The HEK 293T cell line was from the American type culture collection (ATCC, Manassas, VA, USA) and was maintained in Iscove-modified Dulbecco media (IMDM, Gibco, Life Technologies, France) supplemented with 10% foetal calf serum (FCS, Gibco, Life technologies, France) and 20 µg/mL gentamycin (Gibco, Life Technologies, France). The T2 cell line was from ATCC and was maintained in Roswell Park Memorial Institute medium 1640 (RPMI 1640) Glutamax (Gibco, Life Technologies, Courtaboeuf, France) supplemented with 10% FCS, 1.2% nonessential amino acids, 0.2% gentamycin, 0.12% β-mercaptoethanol (all from Gibco, Life Technologies, Courtaboeuf, France), and 1.2% sodium-pyruvate (Sigma-Aldrich, Saint-Quentin Fallavier, France). For activation experiments, PDC*line cells were cultured with Toll-like receptor-7 (TLR7) ligand R848 (1 µg/mL, Invivogen, Toulouse, France) or retroviral supernatant (RVSN, prepared as described in Section 2.2) for 48 h before measuring CD40 expression by flow cytometry (Phycoerythrin-conjugated anti-CD40; Beckman Coulter) and interleukin 6 (IL-6) and tumour necrosis factor α (TNF-α) secretion in the supernatant via the cytometric bead array method (CBA) from Becton Dickinson (BD Biosciences, Le Pont de Claix, France) using the protocol provided by the manufacturer.

The following peptides were purchased from Polypeptide: Melan-A_26–35_ A27L (ELAGIGILTV), gp100_209–217_ (IMDQVPFSV), gp100_471–480_ (VPLDCVLYRY), tyrosinase _369–377_ (YMDGTMSQV), Mage-A3_271–279_ (FLWGPRALV), influenza virus (Flu) M1_58–66_ (GILGFVFTL), cytomegalovirus (CMV) pp65_495–503_ (NLVPMVATV), CMVpp65_417–426_ (TPRVTGGGAM), Epstein-Barr virus (EBV) BMLF1_280–288_ (GLCTLVAML), and Human Immunodeficiency virus (HIV) gag_77–85_ (SLYNTVATL).

### 2.2. Design of Constructs, Generation of Retrovirus, and Transduction of PDC*Line

CMVpp65 and tyrosinase genes were kindly provided by Jenifer MacIntosh and Amit Jathoul (University College London, London, UK). Gp100, Melan-A_A27L_, Mage-A3, and Lysosome-associated membrane protein-1 (LAMP-1) sequences were purchased from GeneArt (Thermofischer Scientific, Illkrich, France). The HLA-B*35:01-pcDNA3.3 was kindly provided by N. Labarrière and H. Benlalam (CRCINA, INSERM, Université d’Angers, Université de Nantes, Nantes, France). These genes were cloned in an optimized SFG vector containing a ∆CD34 molecule as a marker allowing the easy sorting of transduced cells using anti-CD34 microbeads (Miltenyi Biotec, Paris, France), as previously described [18]. Two categories of polyepitopes were developed using one occurrence of four HLA-A*02:01 peptides (Table 1). The ‘memory/naïve’ polyepitope was composed of peptides from Flu, EBV, and CMV antigens, activating the memory response, and from the Melan-A_A27L_ antigen, activating the naïve immune response. The tumour polyepitope contained peptides from four melanoma antigens: Mage-A3, tyrosinase, gp100, and Melan-A_A27L_. To take into account the role of the seven N-terminal amino acids in the natural processing of the peptides presented in HLA-A*02:01 molecules [19], we designed two types of polyepitopes with short peptides (9–10 amino acids (aa)) and long peptides (16–17 aa). Then, the order of peptide was chosen using SYFPEITHI software (http://www.syfpeithi.de/, accessed on 8 February 2021) with the aim of avoiding combinatory epitopes. In total, four polyepitope constructs were made, as presented in Table 1. Polyepitope constructs were generated using overlap PCR as previously described [20] and cloned into the SFG vector containing a Kozak sequence for optimal expression.

RD114-pseudotyped retroviral supernatants (RVSNs) were generated by transient transfection of HEK 293T cells with the retroviral vector and Peq-Pam plasmid (Moloney GagPol, kindly provided by M. Pule, Cancer Institute, University College London, London, UK) and RDF plasmid (RD114 envelope plasmid, kindly provided by M. Pule, Cancer Institute, University College London, London, UK) using GenJuice (Novagen, Sigma-Aldrich, Saint-Quentin Fallavier, France) as previously described [21]. PDC*line cells were then transduced with viral supernatant using retronectin (Takara Bio Europe, Saint-Germain-en-Laye, France), and CD34 expression was assessed by flow cytometry (Allophycocyanin-conjugated anti-CD34; Becton Dickinson Biosciences, Le Pont de Claix, France) to determine the efficacy of transduction (50% to 90%). When needed, the transduced cells were sorted with a CD34 microbead kit (Miltenyi Biotec, Paris, France). Gene expression was stable for at least three months.

### 2.3. Detection of Peptide Presentation Using Specific Clones

For this assay, we used the following HLA-A*02:01-specific T-cell clones recognizing Mage-A3_271–279_ (PENA clone), tyrosinase _369–377_ (WOLF clone), and gp100_209–217_ (DYMA clone) kindly provided by Pierre Coulie (LICR, Bruxelles, Belgium), and Melan-A_26–35L_ (24B7) kindly provided by Brigitte Dréno (UTCG, Nantes, France). HLA-B*35:01-specific T-cells recognizing gp100_471–480_ were kindly provided by Houssem Benlalam [22]. Briefly, 25,000 T-cell clones were stimulated with transduced or peptide-loaded PDC*line cells in RPMI Glutamax medium (Gibco, Life technologies, France) supplemented with 10% FCS (Gibco, Life technologies, France) and IL-2 (10 U/mL; Proleukin, Novartis, Basel, Switzerland) in a 96-well plate. As a positive control, T-cell clones were stimulated with 10 ng/mL phorbol myristate acetate (PMA, Sigma-Aldrich, Saint-Quentin Fallavier, France) and 1 µg/mL ionomycin (Sigma-Aldrich, Saint-Quentin Fallavier, France). After 20 h of culture, 100 µL of supernatant was removed and stored frozen at −20 °C until use. Interferon γ (IFN-γ) or IL-5 was measured in the supernatant via a cytometric bead array (CBA) method using the manufacturer’s protocol (BD Biosciences, Le Pont de Claix, France).

### 2.4. Priming and Expansion of Antigen-Specific T-Cells

Specific T-cells were expanded using cocultures with the transduced PDC*line or peptide-loaded PDC*line cells. Cultures were performed in RPMI 1640 Glutamax supplemented with nonessential amino acids (Gibco, Life technologies, France), 1 mM sodium pyruvate (Sigma-Aldrich, Saint-Quentin Fallavier, France), 20 µg/mL gentamycin, and 10% FCS (Gibco, Life technologies, France). The non-transduced PDC*line was loaded via a 3 h incubation period with peptides of interest and then washed. Then, 30 Gy irradiated peptide-loaded cells or transduced cells were cocultured with HLA-A*02:01-matched PBMCs or purified CD8^+^ T-cells at a 1:10 ratio in culture medium for seven days. In some conditions, cultures were stimulated again at weekly intervals with the peptide-loaded PDC*line and 200 U/mL IL-2 (Proleukin, Novartis, Switzerland). Specific CD8^+^ T-cell expansion was assessed by multimer labeling of PBMCs initially and at different steps of the culture. Cells were resuspended in Hank’s balanced salt solution (HBSS, Gibco, Life Technologies, France) with 2% FCS and HLA-A2 or HLA-B7 multimers, as well as anti-CD3 (BV421-conjugated, BD Biosciences) and anti-CD8 antibodies (PerCP-Cy5.5-conjugated, BD Biosciences) and then analysed by flow cytometry and FlowJo software (Tree Star, Inc., Ashland, OR, USA). Either tetramers (ITag, Beckman Coulter, Villepinte, France) or dextramers (Immudex, Denmark) were used as multimers.

### 2.5. Cytotoxicity Assay

Antigen-specific cytotoxic activity was measured by performing a standard ^51^Cr release assay. CD8^+^ effector T-cells were sorted from the coculture using an EasySep human T-cell enrichment kit (Stem Cell Technologies, Grenoble, France). Peptide-loaded ^51^Cr-labeled T2 cells were plated with effector T-cells at the E:T ratio of 1:1 or 10:1 in U-bottom 96-well plates. After 4 h of incubation, the radioactivity contained in 30 µL of supernatant was measured on a microplate scintillation counter Top Count NXT (Perkin Elmer, Shelton, CT, USA). The mean of triplicate measurements was expressed as a percentage of specific lysis using the following formula: (sample release − spontaneous release)/(maximal release − spontaneous release) × 100.

### 2.6. Statistical Analysis

Statistical analyses were performed using GraphPad Prism software (GraphPad Software, v5.01, San Diego, CA, USA). Nonparametric Friedman or Kruskal–Wallis tests were used, with Dunn’s multiple comparison post hoc test.

## 3. Results

### 3.1. The Transduction Process Does Not Activate PDC*Line Cells

**Figure 1:**
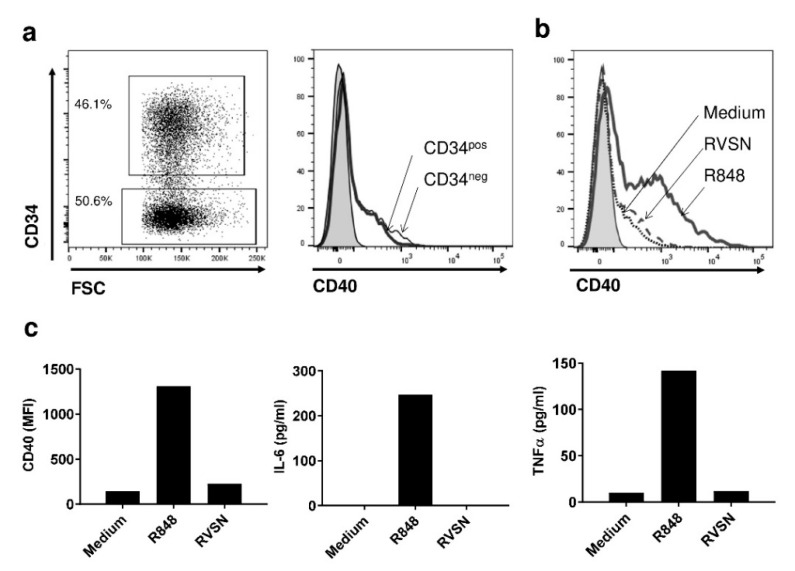
The transduction process does not activate human HLA-A*02:01 plasmacytoid dendritic cell line (PDC*line). (**a**) PDC*line cells were transduced with retroviral supernatant (RVSN) for 48 h and labelled with anti-CD40 antibody. The CD34-positive and -negative populations were gated, and CD40 expression was evaluated in the CD34-positive (CD34pos, thick line) and CD34-negative population (CD34neg, thin line) of cells. (**b**,**c**) PDC*line cells were treated or not with R848 or virus supernatant for 48 h before CD40 labelling as in (**a**). Percentage of positive cells (**b**) or mean fluorescence intensity (MFI) (**c**) are represented. Gray-filled histograms correspond to control isotype labelling. In addition, interleukin 6 (IL-6) and tumour necrosis factor α (TNF-α) were measured in the cell culture supernatant via cytometric bead array (CBA) (**c**).

Following retroviral transduction, the expression of the CD34 transgene-associated marker generally reached 80%, demonstrating that the transduction efficacy of PDC*line cells using retrovirus was very good. In the rare cases where the percentage of transduction was lower, an enrichment of transduced cells was performed using a CD34-positive selection kit. Interestingly, the gene expression was stable for several months (not shown). As PDC*line cells are derived from plasmacytoid dendritic cells known to express TLR7 and TLR9, they could be activated by the single-strand RNA viruses of the retrovirus constructs used to transduce them. Thus, we evaluated the consequences of the transduction process on the activation of PDC*line cells by measuring upregulation of activation markers and cytokine production. Interestingly, using the CD34 marker, it was also possible to distinguish transduced from non-transduced PDC*line cells and to measure the expression of the CD40 activation marker on both cell subsets using flow cytometry (Figure 1a). Results clearly showed no difference in CD40 expression by transduced (CD34^pos^) or non-transduced (CD34^neg^) cells, in comparison with TLR7 ligand (R848) activation (Figure 1b,c). This was confirmed by the observation that no cytokine secretion was detected in the culture medium in the presence of retroviral supernatant (RVSN; Figure 1c). Thus, RVSN did not lead to the activation of PDC*line cells.

### 3.2. The Transduction of Memory/Naïve Polyepitope Gene in PDC*Line Cells Induces Multi-Specific T-Cell Responses

Until now, the potency of the PDC*line as antigen-presenting cells was evaluated using peptides that were passively loaded on the cells [11,12,13,14]. To favour internal peptide processing, we decided to design a PDC*line endogenously expressing antigens of interest by viral transduction. We first evaluated the processing and presentation of immunogenic peptides by PDC*line cells transduced by a memory/naïve polyepitope gene. Thus, we designed a polyepitope construct (ICEM, Table 1) by combining four well-known HLA-A*02:01 epitopes derived from three viral proteins (memory antigens: influenza M1, CMV pp65, and EBV BMLF1) and one tumour protein (naïve antigen: Melan-A) presenting a high basal precursor frequency in healthy donors. Two constructs taking into account the consensus sequence of each peptide and the same sequence flanked with the seven natural amino acids located in the N-terminal position were used (ICEM-S (short) and ICEM-L (long), respectively; Table 1). Indeed, Schatz et al. showed that the final N-terminus of the Major histocompatibility complex (MHC) class I ligand could be important for peptide processing, as it is the substrate for cytosolic and endoplasmic reticulum aminopeptidases [19]. Moreover, the order of peptides was defined in order to avoid combinatory epitopes.

**Figure 2:**
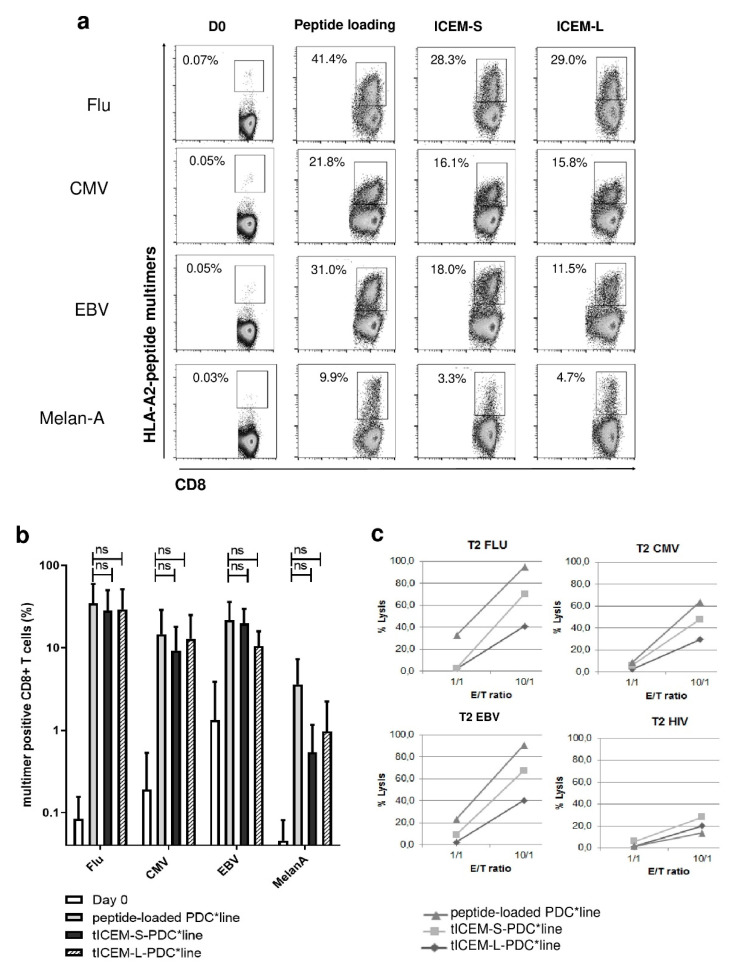
The transduction with memory/naïve polyepitope constructs induced multi-specific and functional T-cells. Peptide-loaded PDC*line cells or PDC*line cells transduced with memory/naïve polyepitopes were cocultured with peripheral blood mononuclear cells (PBMCs) from five healthy donors. Two ICEM polyepitope constructions were used differing by peptide length (S: short; L: long). ICEM indicates the order of HLA-A2-restricted peptides (influenza M1, CMV pp65, EBV-BMLF1, and Melan-A). Antigen-specific T-cell expansion was measured following a 14-day culture using HLA-A2/peptide multimer staining on CD3^+^ CD8^+^ cells. In (**a**)**,** representative dot plots are shown, and (**b**) represents the results obtained following coculture with the five different donors. Values inside the dot plots indicate the percentage of specific T-cells. In (**c**), the functionality of expanded specific T-cells was evaluated and shown. Following expansion, specific T-cells were submitted to a ^51^Cr cytotoxic assay using peptide-loaded T2 target cells at two different effector/target ratios (1:1 and 10:1). Cytotoxicity against Flu, CMV, and EBV peptide-loaded T2 target cells was measured following 4 h of incubation. Human immunodeficiency virus (HIV) peptide-loaded cells were used as a negative control. Statistics are based on one-way ANOVA (nonparametric Friedman test with Dunn’s post hoc test; ns: nonsignificant).

The expansion of peptide-specific CD8^+^ T-cells was then evaluated by performing 14-day cocultures of PBMCs from healthy donors (*n* = 5) and the memory/naïve polyepitope-transduced irradiated PDC*line. In parallel, standard cocultures with individual peptide-loaded PDC*line cells were conducted. As shown in Figure 2a,b, PDC*line cells transduced by either the short (ICEM-S) or the long (ICEM-L) forms of polyepitopes led to a substantial expansion of T-cells specific to the four peptides similarly to peptide-loaded PDC*line cells. Huge expansions of CMV-, BMLF1-, and Flu peptide-specific T-cells were observed. Generally, frequencies greater than or equal to 10% were obtained, meaning that, in the same culture with transduced PDC*line, around 30% of CD8^+^ T-cells were specific for a viral antigen. Interestingly, an expansion of Melan-A-specific T-cells was also found, indicating that the transduced PDC*line was also able to prime and expand naïve T-cells simultaneously to the activation and expansion of memory antigen-specific T-cells.

We then assessed the cytotoxic activity of the peptide-specific T-cells expanded. FluM1_58–66_, CMVpp65_495–503_, or EBV BMLF-1_280–288_-loaded T2 cells were used as targets in ^51^Cr release cytotoxic assays. As expected, the CD8^+^ T-cells expanded in coculture with single-loaded PDC*line cells displayed a high and specific lytic activity against FluM1-, CMV-, or EBV BMLF-1-loaded target cells (Figure 2c). Similarly, peptide-specific CD8^+^ T-cells generated by coculture with polyepitope-transduced PDC*line cells displayed a specific and a strong cytotoxic activity whatever the length of the polyepitope.

Thus, a transduced memory/naïve polyepitope was efficiently produced and processed by PDC*line cells, resulting in concomitant functional presentation of the encoded peptides in the context of the same HLA molecule in the same culture, allowing the simultaneous expansion of functional multi-specific CD8^+^ T-cells.

### 3.3. Tumour Polyepitope or Whole-Tumour Antigen Gene-Transduced PDC*Line Cells Allow the Activation and Priming of Multi-Tumour Antigen-Specific T-Cell Responses

We next addressed the question whether PDC*line cells, as potent antigen-presenting cells, could process and present peptides derived from several tumour antigens endogenously expressed, and then prime and expand naïve antigen-specific T-cells. We decided to use tumour antigens derived from melanoma as a cancer model and to transduce the PDC*line cells by retroviruses encoding either whole antigenic proteins or a polyepitope. We used four constructs encoding the whole proteins Mage-A3 (M3), tyrosinase (T), gp100 (G), and Melan-A (Me) or one polyepitope construct encoding HLA-A*02:01-restricted peptides expressed by these four proteins (M3TGMe). Two polyepitope constructions with short or long sequences were made (M3TGMe-S and M3TGMe-L; Table 1). Peptide presentation by the transduced PDC*line cells was evidenced by the cytokine production of HLA-A2-restricted antigen-specific T-cell clones.

**Figure 3:**
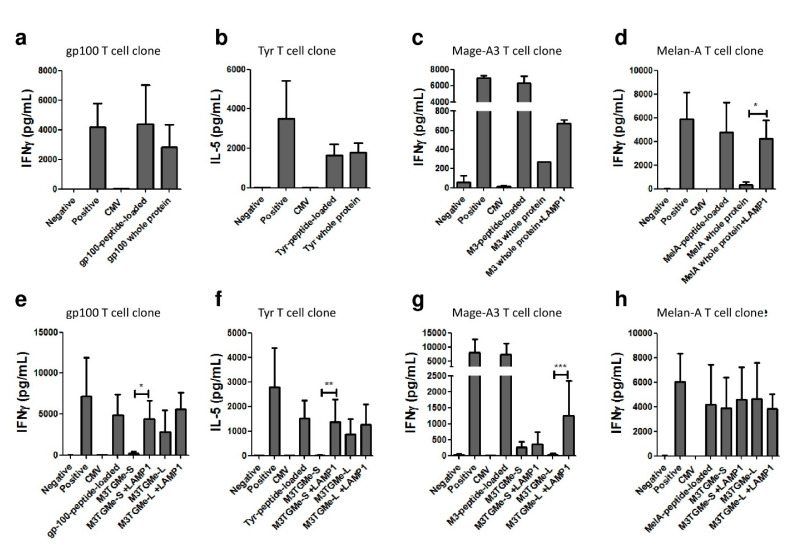
PDC*line cells transduced with tumour polyepitope or whole-tumour antigen constructs induce multi-specific T-cell responses. Peptide-loaded or transduced PDC*line cells were cocultured with HLA-A2/peptide-specific T-cell clones (anti-Mage-A3, anti-gp100, anti-tyrosinase, and anti-Melan-A) for 20 h and interferon γ (IFN-γ) or IL-5 production was measured in cell culture supernatant to measure the T-cell response. (**a**–**d**) T-cell clones were cultured with PDC*line transduced with genes encoding the whole protein or (**e**–**h**) with PDC*line transduced with M3TGMe-S or M3TGMe-L polyepitope constructs. When indicated, the lysosome-associated membrane protein-1 (LAMP-1) transmembrane and C-terminal sequence was added to the construct. CMV peptide-transduced PDC*line cells and tumour peptide-loaded PDC*line cells were used as negative and positive controls of specificity, respectively. Nonactivated or phorbol myristate acetate (PMA)/ionomycin-treated T-cells were used as negative or positive controls of the assay, respectively. The mean +SD of at least four experiments is shown. Statistics are based on the Kruskal–Wallis test; * *p* < 0.05, ** *p* < 0.02, and *** *p* < 0.01.

Considering whole proteins, our results show that PDC*line cells transduced by the construct encoding the proteins gp100 and tyrosinase were able to present tumour peptides to T-cell clones as efficiently as the peptide-loaded PDC*line (Figure 3a,b). By contrast, there was no or very low presentation of peptides derived from Mage-A3 or Melan-A by transduced PDC*lines cells (Figure 3c,d). We hypothesized that co-transduction with the transmembrane and C-terminal sequence of LAMP-1 could improve the presentation of these antigens. Indeed, due to its ability to address proteins to endolysosomal compartments, it has been described that LAMP-1 participates in antigen processing and presentation by MHC I and II [23]. As shown in Figure 3c,d, the presentation of Mage-A3 and Melan-A peptides was restored by adding the LAMP-1 sequence into the constructs.

When PDC*line cells transduced by the short or long polyepitopes (M3TGMe-S and M3TGMe-L) were used to stimulate T-cell clones, different patterns of response were observed. Indeed, in the case of Melan-A, a similar secretion of IFN-γ by the specific T-cell clone was observed for both constructs (Figure 3h), indicating a good presentation of the peptides derived from the endogenous cell processing of the polyepitope whatever the length of the peptide. By contrast, for Mage-A3 peptide, no cytokine secretion was observed whatever the construct (Figure 3g). Interestingly, in the cases of gp100 and tyrosinase, only the long form of the peptides elicited T-cell activation (Figure 3e,f), suggesting a role for the N-terminal amino acids in their processing as proposed by Schatz et al. Again, in order to improve the processing and the presentation of transduced peptides by PDC*line cells, the transmembrane and C-terminal sequence of LAMP-1 was added to the polyepitope constructs. Interestingly, LAMP-1 addition led to the correct presentation of the short form of gp100 and tyrosinase peptides from the M3TGMe-S-LAMP-1 construct (Figure 3e,f). For Mage-A3 peptide, only the long form of the construct in presence of LAMP-1 elicited correct T-cell activation (Figure 3g).

Altogether, these results show that PDC*line cells, following transduction with whole-tumour proteins or polyepitopes, can efficiently produce, process, and present the tumour peptides to antigen-specific T-cells, with this processing being improved by the addition of the LAMP-1 transmembrane and C-terminal sequence.

**Figure 4:**
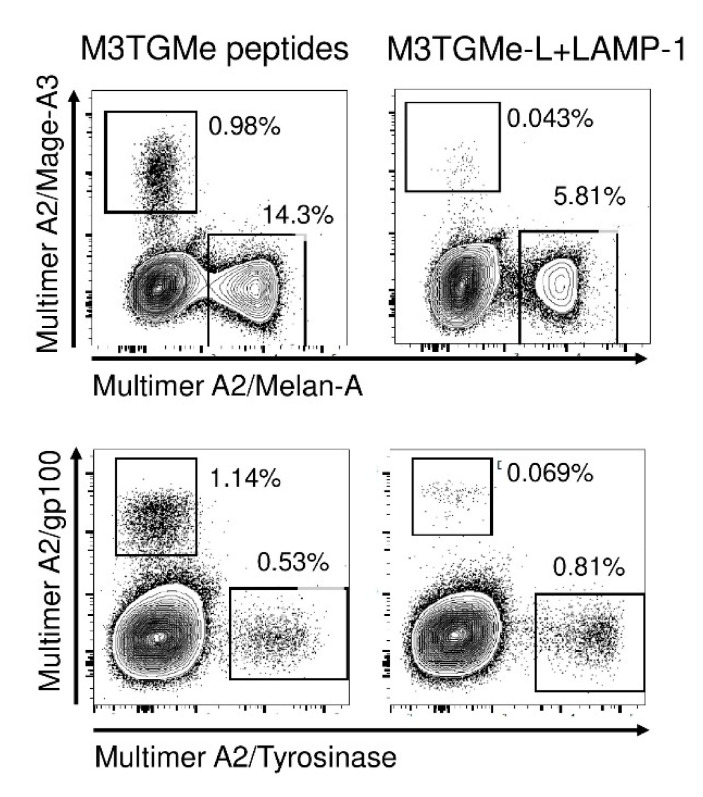
PDC*line cells transduced with tumour polyepitope constructs prime and expand multi-specific T-cells. PDC*line cells were loaded individually with each of the four peptides of interest (Mage-A3, tyrosinase, gp100, or Melan-A peptide) and then mixed together or transduced with the construct encoding the long tumour peptides and LAMP-1 (M3TGMe-L-LAMP-1). Loaded or transduced cells were cocultured with CD8^+^ T-cells purified from healthy donor PBMCs in the presence of IL-2 for 21 days. At the end of the culture, the percentage of peptide-specific CD8^+^ T-cells was assessed using HLA-A2/peptide multimer staining.

In order to demonstrate that PDC*line cells transduced by the tumour polyepitope were able to not only activate T-cell clones, but also prime and expand naïve T-cells, we cocultured them with CD8^+^ T-lymphocytes purified from healthy donor PBMCs, focusing on the response against melanoma-derived antigens. Indeed, T-cells specific for these antigens in healthy donors are naïve [24]. In Figure 4, assessment of T-cells specific for peptides derived from Mage-A3, tyrosinase, gp100, and Melan-A was made after a 21-day coculture of CD8^+^ T-cells with either M3TGMe-L-LAMP-1-transduced or peptide-loaded PDC*line cells. Strikingly, at the end of culture, specific T-cells directed toward all four antigens were detected in both cases, albeit at different levels. Generally, the frequencies obtained in the coculture stimulated with a mix of peptide-loaded PDC*line cells were higher than those in the cocultures with transduced cells. This could be due, in the latter case, to the fact that the peptides from polyepitopes are presented concomitantly by the same PDC*line cells, which was not the case in the peptide-loaded condition.

### 3.4. Efficient Antigen Presentation by PDC*Line Cells via Diverse HLA Molecules Naturally Expressed or Transduced

With the aim of using PDC*line for research or clinical application for other patients than those expressing HLA-A*02:01, we wondered whether the other HLA class I molecules naturally expressed by the cell line were functional and whether PDC*line cells could be engineered to express other HLA molecules of interest.

**Figure 5:**
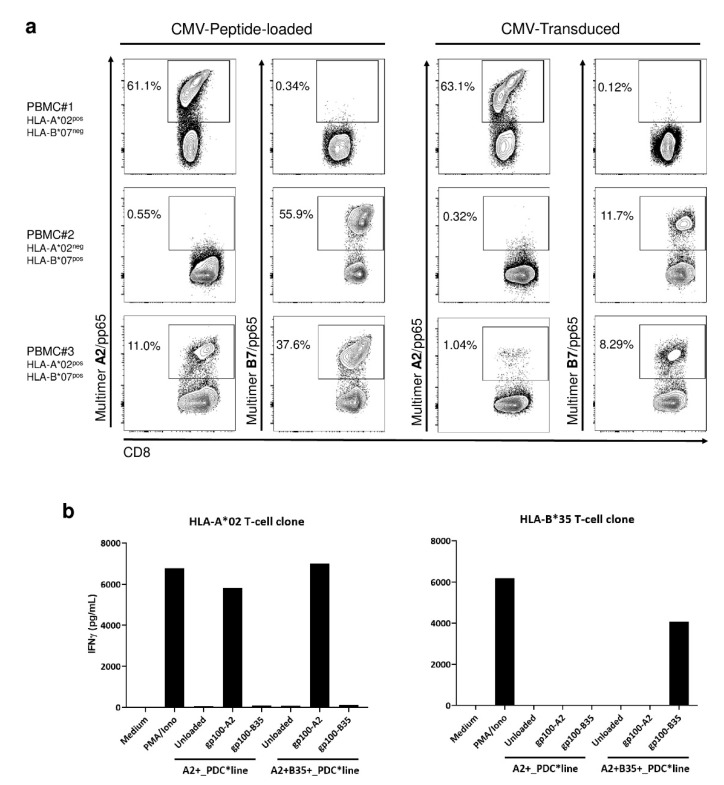
Efficient antigen presentation by PDC*line cells via diverse HLA molecules naturally expressed or transduced. (**a**) PDC*line cells were loaded with HLA-A2- or HLA-B7-restricted CMVpp65-derived peptides or transduced with the whole CMVpp65 gene. Loaded or transduced cells were cocultured with healthy donor PBMCs expressing HLA-A*02 (PBMC#1), HLA-B*07 (PBMC#2), or both molecules (PBMC#3). Antigen-specific CD8^+^ T-cell expansion was measured following a 14-day culture using HLA-A2/peptide or HLA-B7/peptide multimer staining on CD3^+^ CD8^+^ cells. Values inside the dot plots indicate the percentage of specific CD8^+^ T-cells. (**b**) PDC*line cells transduced or not with HLA-B*35:02 gene were loaded with HLA-A*02- or HLA*B35-restricted gp100 peptides and cocultured with the corresponding gp100-specific T-cell clone. After 20 h, IFN-γ was measured in the supernatant via CBA.

As PDC*line cells express naturally HLA-B*07:02, in addition to HLA-A*02:01, we asked if HLA-B7 was functional and if the cells could activate T-cells specific for A2- and B7-restricted antigens at the same time. The cells were transduced with the whole CMV pp65 protein that contains several immunogenic epitopes, including one presented in the context of HLA-A*02:01 (NLVPMVATV) and another one in the context of HLA-B*07:02 (TPRVTGGGAM). Transduced PDC*line cells were compared to peptide-loaded cells for their ability to expand peptide-specific T-cells in cocultures. We used PBMCs from three healthy donors, expressing HLA-A*02, HLA-B*07, or both molecules. As shown in Figure 5a, pp65-transduced PDC*line cells induced HLA-A*02- and/or HLA-B*07-specific CD8^+^ lymphocytes. This expansion is HLA-restricted, as antigen-specific T-cells were expanded only when there was a match between the HLA molecules expressed by the PBMCs and the PDC*line cells. Interestingly, T-cells responding to two epitopes were induced in PBMC#3, positive for both HLA-A*02 and HLA-B*07.

Then, we evaluated the ability of PDC*line cells transduced with a new HLA molecule, HLA-B*35:01, to present peptide derived from gp100 by using specific T-cell clones. In Figure 5b, activation of HLA-A2-restricted or HLA-B35-restricted T-cell clones specific for gp100 was evaluated in cocultures with peptide-loaded PDC*line transduced or not by HLA-B35 molecule. Interestingly, the HLA-B35 T-cell clone was activated only by the HLA-B35-transduced PDC*line cells loaded with HLA-B35-restricted peptide, demonstrating the functionality of this newly expressed HLA molecule at the surface of PDC*line cells. In addition, HLA-B35-transduced PDC*line cells retained their ability to present the HLA-A2-restricted peptides to the HLA-A2 T-cell clones with the same efficacy as the non-transduced cell line.

Thus, we have demonstrated that the PDC*line can be engineered to express antigens that can be presented by all HLA molecules already expressed by the PDC*line or to express HLA molecules of interest, allowing us to broaden the use of this cell line for research or clinical applications.

## 4. Discussion

Because dendritic cells (DC), as professional antigen-presenting cells, are crucial to prime and expand antigen-specific CD8^+^ T-cells, several strategies aim to use or target DCs to induce protective immunity in prophylactic or therapeutic vaccines for infectious diseases or cancer [25]. However, until now, myeloid DCs that were primarily used in human therapy have not shown strong clinical benefit [2,3,26]. Despite their potency to induce an immune response in infectious diseases and cancer [27,28], plasmacytoid DCs have seldom been used in humans due to the difficulty in isolating or producing them in large numbers. The possibility of using a cell line of PDC origin represents, in this context, a great opportunity to develop new potent DC-based vaccines.

We have generated several HLA-A*02:01^pos^-cell lines derived from a patient having a PDC leukaemia [8,9,10]. These cell lines shared the same potency to activate or prime memory or naïve antigen-specific T-cells in viral or tumour models [10,11,12,13,14]. The PDC cell line used here was specifically generated for developing an allogeneic PDC platform for clinical use allowing the design of various peptide-loaded PDC drug products depending on the disease to treat. As an example, one drug product has already been used for the treatment of HLA-A*02:01^pos^ melanoma patients (NCT01863108) in a first-in-human phase I clinical trial [10], and another is currently being evaluated in the treatment of HLA-A*02:01^pos^ lung cancer patients (NCT03970746) in a phase I/II trial.

The aim of this study was to optimize this PDC vaccine platform to facilitate and lower the cost of manufacturing and to make this treatment available for a larger number of patients. Thus, we evaluated the possibility to engineer this PDC*line to obtain new PDC cell lines endogenously expressing either viral or tumour antigens to avoid the peptide loading step or displaying new HLA class I molecules to enlarge the target patient population.

Lentiviral approaches have been largely used for engineering myeloid dendritic cells [29]. In the present study, even though lentiviruses were shown previously to transduce PDCs [30], the use of retroviruses was preferred as PDC*line cells have a strong proliferative property. The feasibility of the retroviral transduction process was demonstrated to be efficient as, generally, more than 80% of cells were routinely efficiently transduced. Interestingly, thanks to the inclusion of the CD34 marker, the cells of interest can easily be identified and potentially enriched with a clinical-grade magnetic cell sorter in cases of lower transduction efficacy, as proposed elsewhere [31]. Moreover, the transduction process did not affect the features of the cells, as they did not display an activation phenotype afterward.

Thanks to the use of polyepitope genes encoding viral or tumoral peptides, we have shown that the transduced PDC*line was able to process the peptides that are endogenously expressed and to present them to peptide-specific T-cells with great efficiency. In addition, the specificity and the functionality of the effector cells were confirmed using a cytotoxicity assay. As all the transduced peptides were expressed by the same cells, multiple specific T-cells were generated using the same stimulating cells, representing an added value of the platform. However, the design of the peptide sequences used in the viral construction can impact its processing and presentation as suggested by others [19]. It was decided, in this study, to evaluate the impact of the addition of seven N-terminal amino acids to the natural MHC ligands. Indeed, for some peptides, we observed that this additional sequence was needed to get a good presentation. This is in agreement with the results obtained when the whole protein was transduced, as the corresponding peptides were well presented. However, for Mage-A3 and Melan-A proteins or peptides, poor antigen presentation was observed even in the presence of the additional sequence. Indeed, it has been described that intracellular protein degradation mechanisms interfered with the correct peptide presentation for these two proteins [32,33].

Interestingly, the poor peptide presentation by transduced PDC*line cells observed for some antigens with the constructs encoding the polyepitopes (Mage-A3, short or long form) or the whole protein (Mage-A3, Melan-A) was reversed by adding the late endosomal-addressing LAMP-1 sequence. In a previous report, Bonehill et al. demonstrated that the use of such targeting sequences greatly improved the presentation of tumour peptide in the context of the HLA class I molecule [34]. This addition did not modify the presentation of the other peptides that were already well presented alone, suggesting that LAMP-1 sequences should be added systematically for designing new constructs to ensure the efficient presentation of all peptides contained in polyepitopes or whole proteins.

Of particular interest in this study, we showed that the PDC*line transduced with the optimized construct (M3TGMe-L-LAMP-1) can prime and expand antigen-specific T-cells from healthy donor CD8^+^ T-cells in a similar way to the individually peptide-loaded PDC*line, demonstrating the potency of the platform in terms of antigen presentation ability.

We next demonstrated that gene engineering enabled adapting the PDC*line cell technology to also treat patients that do not express HLA-A*02:01. Indeed, HLA-B*07 already expressed by the PDC*line was also functional in presenting and expanding HLA-B*07-restricted peptide-specific T-cells. More strikingly, PDC*line cells can be transduced with other HLA molecules of interest, leading to the activation of the matched antigen-specific T-cells without needing to delete the naturally expressed HLA molecules. In addition, transduction did not affect the presentation of peptide by natural HLA molecules.

Despite the fact that the PDC*line platform has shown in human (melanoma patients, [10]) its potency to prime and expand antigen-specific T-cells, it would be interesting to evaluate if the transduced cells show the same activity in an in vivo model as in vitro. As PDC*line cells are from human origin, these types of experiments required an HLA-matched humanized mouse model displaying a mature and naïve human immune system. Unfortunately, although such models have been recently developed, they are not fully mastered to be used for such a comparison.

All these results demonstrate the ease to virally engineer the PDC*line with retrovirus, providing a stable expression of the transgene. Through introduction of the whole protein, all HLA class I epitopes of this protein are available for the presentation of these peptides by the PDC*line, as demonstrated with the CMVpp65 protein. In addition, genes encoding the peptides of interest thanks to a polyepitope construct could be used to prime and induce expansion of antigen-specific T-cells directed against different viral proteins for the same or different viral or tumour proteins to avoid the escape from the immune system. In addition, by demonstrating that other HLA class I molecules can be added to the cell line, we showed that this approach is not restricted to only HLA-A*02:01 or HLA-B*07:02 patients.

Viral transductions are now well mastered at the clinical level, and retroviral vectors have been widely used for clinical applications in cancer and infectious diseases [29]. In addition, we have shown that lentiviruses can be used with similar high transduction efficiency [30].

The demonstration of the effective viral transduction of PDC*line cells strengthens and broadens the scope of the PDC*line platform for use in adoptive or active immunotherapy for the treatment of infectious or cancer diseases. As we demonstrated the potency of this PDC*line as a professional APC in this study and elsewhere [10,11], the simplicity of the manufacturing process compared to other platforms [35] renders this technology very attractive for priming/activation and expansion of antigen-specific T-cells both in vitro and in vivo for clinical use in many diseases.

## 5. Conclusions

The demonstration of the effective viral transduction of PDC*line cells strengthens and broadens the scope of the PDC*line platform for use in adoptive or active immunotherapy for the treatment of infectious or cancer diseases. As we demonstrated the potency of this PDC*line as a professional antigen-presenting cell in this study and elsewhere [10,11], the simplicity of the manufacturing process compared to other platforms [35] renders this technology very attractive for priming/activation and expansion of antigen-specific T-cells both in vitro and in vivo for clinical use in many diseases.

## Figures and Tables

**Table 1:** Names, lengths and sequences of polyepitopes used in the study.

Name *	Length (aa)	Sequence **
ICEMe-S	37	gILGFVFTLnLVPMVATVgLCTLVAMLeLAGIGILTV
ICEMe-L	65	iLSPLTKgILGFVFTLqAGILARnLVPMVATVmQAIQNAgLCTLVAMLhSYTTAEeLAGIGILTV
M3TGMe-S	37	fLWGPRALVyMDGTMSQViMDQVPFSVeLAGIGILTV
M3TGMe-L	65	sDPACYEfLWGPRALVmHNALHIyMDGTMSQVhSSSAFTiMDQVPFSVhSYTTAEeLAGIGILTV

* I: influenza M1; C: Cytomegalovirus (CMV) pp65; E: Epstein-Barr virus (EBV) BMLF-1; Me: Melan-A; M3: Mage-A3; T: tyrosinase; G: gp100; L: long; S: short. ** Lowercase letters indicate the beginning of each peptide sequence.

## Data Availability

No new data were created or analyzed in this study. Data sharing is not applicable to this article.
